# Novel Alleles of Phosphorus-Starvation Tolerance 1 Gene (*PSTOL1*) from *Oryza rufipogon* Confers High Phosphorus Uptake Efficiency

**DOI:** 10.3389/fpls.2017.00509

**Published:** 2017-04-11

**Authors:** Kumari Neelam, Shiwali Thakur, Neha, Inderjit S. Yadav, Kishor Kumar, Salwinder S. Dhaliwal, Kuldeep Singh

**Affiliations:** ^1^School of Agricultural Biotechnology, Punjab Agricultural UniversityLudhiana, India; ^2^Department of Soil Science, Punjab Agricultural UniversityLudhiana, India; ^3^ICAR-National Bureau of Plant Genetic ResourcesNew Delhi, India

**Keywords:** rice (*Oryza sativa L*.), phosphorus uptake efficiency, *Oryza rufipogon*, allele mining, phosphorus starvation tolerance 1 (*PSTOL1*), SSR markers

## Abstract

Limited phosphorus availability in the soil is one of the major constraints to the growth and productivity of rice across Asian, African and South American countries, where 50% of the rice is grown under rain-fed systems on poor and problematic soils. With an aim to determine novel alleles for enhanced phosphorus uptake efficiency in wild species germplasm of rice *Oryza rufipogon*, we investigated phosphorus uptake1 (*Pup1*) locus with 11 previously reported SSR markers and sequence characterized the phosphorus-starvation tolerance 1 (*PSTOL1*) gene. In the present study, we screened 182 accessions of *O. rufipogon* along with Vandana as a positive control with SSR markers. From the analysis, it was inferred that all of the *O. rufipogon* accessions undertaken in this study had an insertion of 90 kb region, including *Pup1*-K46, a diagnostic marker for *PSTOL1*, however, it was absent among *O. sativa* cv. PR114, PR121, and PR122. The complete *PSTOL1* gene was also sequenced in 67 representative accessions of *O. rufipogon* and Vandana as a positive control. From comparative sequence analysis, 53 mutations (52 SNPs and 1 nonsense mutation) were found in the *PSTOL1* coding region, of which 28 were missense mutations and 10 corresponded to changes in the amino acid polarity. These 53 mutations correspond to 17 haplotypes, of these 6 were shared and 11 were scored only once. A major shared haplotype was observed among 44 accessions of *O. rufipogon* along with Vandana and Kasalath. Out of 17 haplotypes, accessions representing 8 haplotypes were grown under the phosphorus-deficient conditions in hydroponics for 60 days. Significant differences were observed in the root length and weight among all the genotypes when grown under phosphorus deficiency conditions as compared to the phosphorus sufficient conditions. The *O. rufipogon* accession IRGC 106506 from Laos performed significantly better, with 2.5 times higher root weight and phosphorus content as compared to the positive control Vandana. In terms of phosphorus uptake efficiency, the *O. rufipogon* accessions IRGC 104639, 104712, and 105569 also showed nearly two times higher phosphorus content than Vandana. Thus, these *O. rufipogon* accessions could be used as the potential donor for improving phosphorus uptake efficiency of elite rice cultivars.

## Introduction

Rice (*Oryza sativa* L.), one of the major staple food crops in the world, is critical to food security for billions of people around the world. Calories from rice are particularly important in Asia, especially among the poor, where it accounts for 50–80% of the daily calorie intake (http://www.gramene.org/). The estimated demand for rice in India is projected to go up to 121.2 million tons by the year 2030, 129.6 by the year 2040 and 137.3 million tons by the year 2050 as compared to 90–104 million tons being produced currently (http://www.crri.nic.in/ebook_crrivision2050_final_16Jan13.pdf). This indicates that rice production needs to be increased by 32% in the next 33 years for fulfilling the internal consumption of India. Keeping in view the situation when the area growth rate is negative and decreasing at the rate of 0.15% per year under rice, utilization of poor and problematic soils for sustaining yield requirement is one of the most promising ways.

Rice requires phosphorus to survive and thrive. It is a key element in plant metabolism, root growth, maturity, and yield. Phosphorus (P) deficiency leads to various physiological disorders in rice such as stunted growth, reduced tillering, thin and spindle stems, reduced number of grains per panicle (http://www.Knowledgebank.irri.org/phosphorus-deficiency) and ultimately leads to the reduction in the yield of rice plants. In Asia, 60% of the rain-fed lowland rice is produced on poor and problem soils that are naturally low in phosphorus or P fixing (Gamuyao et al., 2012). Phosphorus deficiency is widespread in Bangladesh, India, Indonesia, Nepal, Pakistan, South China, and Vietnam (Wissuwa and Ae, 2001; Haefele and Hijmans, 2009). In India, nearly 61.02% of the soils are found low in available P, 25.89 and 13.09% are found medium and high in available P content (Hasan, 1996; Muralidharudu et al., 2011). The hurdle further increases due to the presence of a non-renewable source of phosphatic fertilizers. The indigenous deposits of rock phosphate are barely able to meet 10% of the phosphate fertilizer demand in India. For the rest of the need (90%), India depends on imports of raw materials and processed phosphatic fertilizer products (Sharma and Thaker, 2011). Large quantities of finished products of fertilizer are imported in India every year, along with raw materials and intermediates for producing different fertilizers indigenously. In 2000-01, import of finished products (on N + P_2_O_5_ + K_2_O nutrient basis) was 2.194 million tons, which rose to 12.208 million tons in 2010–11 (Majumdar et al., 2013). Besides, about 5 million tons of rock phosphate and 2 million tons of phosphoric acid are imported every year. The availability of rock phosphate from domestic sources is about 1.86 million tons (Majumdar et al., 2013) which is nearly one by seventh of the total demand. Further, annual outgo on fertilizer subsidy during 2013–14 was Rs. 71,251 crores, out of which Rs. 29,427 crores were shared by phosphatic and potassic fertilizers. Therefore, the development of rice varieties with sustainable productivity under the problematic soil is a valid approach toward reducing the economic burden of the country.

The wild species germplasm of rice constitutes the most important genetic resources for rice improvement. Rice belongs to genus *Oryza* and tribe *Oryzeae* of the family *Gramineae (Poaceae)*. The genus *Oryza* contains 24 recognized species, of which 22 are wild species (Vaughan et al., 2003). The wild species have either 2n = 24 or 2n = 48 chromosomes representing AA, BB, CC, BBCC, CCDD, EE, FF, GG, and HHJJ genomes (Brar and Khush, 2003). Several genes and QTLs have been mined from wild species of rice for resistance to biotic and abiotic stresses and for enhancing the productivity of modern cultivars (Khush et al., 1977; Xiao et al., 1996; Moncada et al., 2001; Aluko et al., 2004; Linh et al., 2008; Rangel et al., 2008; Chen et al., 2009; Khush, 2013). In rice, the low-Pi tolerance is naturally present in wild germplasm/landraces and could be used to improve phosphorus acquisition efficiency (PAE) and phosphorus use efficiency (PUE) in modern varieties (Gamuyao et al., 2012). A major QTL for P-deficiency tolerance was mapped on chromosome 12 (*Pup1*) from the aus type rice variety Kasalath, explaining 70% of the variance (Wissuwa et al., 2002). Among various markers developed by Chin et al. (2010) for marker assisted breeding of phosphorus uptake efficiency, only OsPupK46-2 was found associated with the trait and later named as phosphorus-starvation tolerance 1 (*PSTOL1*) gene by Gamuyao et al. (2012). This gene is absent from the rice reference genome (Nipponbare) and in the genomes of other *indicia* varieties which are susceptible to phosphorus deficiency (Wissuwa et al., 2002). The *PSTOL1* act as an enhancer of early root growth and promotes more phosphorus uptake (Gamuyao et al., 2012). Therefore, it is highly desirable to explore, utilize and transfer new alleles of *PSTOL1* gene to the elite cultivars for improving their yield under low phosphorus soil conditions. Only a few reports are available on allelic diversity present among the rice wild species germplasm for the *PSTOL1* gene (Pariasca-Tanaka et al., 2014; Vigueira et al., 2016). Moreover, all of the breeding programs worldwide for improving phosphorus uptake are focused on the transfer of *PSTOL1* gene from Kasalath (*aus* type) and African rice (*O. glaberrima Steud*), leading to the narrowing of genetic variability. In order to deploy novel genes/alleles for improving phosphorus uptake efficiency, our primary objective is to investigate *Oryza rufipogon* accessions for allelic diversity at *PSTOL1*, its validation under the phosphorus-deficient conditions and further its transfer to elite rice *indica* cultivars.

## Materials and methods

### Plant materials

For SSR marker analysis in this study, 182 *O. rufipogon* accessions from 10 different countries *viz*. Bangladesh (*n* = 8), Cambodia (*n* = 31), Thailand (*n* = 24), Myanmar (*n* = 16), Taiwan (*n* = 4), Vietnam (*n* = 20), Nepal (*n* = 20), Laos (*n* = 8), Papua New Guinea (*n* = 13), and India (*n* = 38) were undertaken. These germplasm accessions were originally procured either from the International Rice Research Institute (IRRI), Philippines or from National Rice Research Institute (NRRI), Cuttack and being actively maintained at Punjab Agricultural University (PAU), Ludhiana. The rice cultivars, Punjab Rice 114 (PR114), Punjab Rice 121 (PR121), Punjab Rice 122 (PR122), and Punjab Basmati 3 (PB3) were used as negative checks. The upland rice variety Vandana was selected as a positive control due to the presence of 90 kb of phosphorus uptake 1 (*Pup1*) locus. The list of accessions undertaken along with their country of origin is given in Supplementary Table S1. Out of 182 accessions, 67 were sequenced for complete coding sequences (CDS) of *PSTOL1* (Table 1).

**Table 1:** **Selected *O. rufipogon* accessions for allele mining at *PSTOL1* gene**.

**S. No**.	**Accessions**	**Country of origin**
1	IRGC 93060	Cambodia
2	IRGC 89223	Cambodia
3	IRGC 89230	Cambodia
4	IRGC 93048	Cambodia
5	IRGC 93059	Cambodia
6	IRGC 105726	Cambodia
7	IRGC 106336	Cambodia
8	IRGC 83804	Cambodia
9	IRGC 86659	Cambodia
10	IRGC 89012	Cambodia
11	IRGC 105569	Cambodia
12	IRGC 93034	Cambodia
13	CR 100488A	India
14	CR 100013	India
15	CR 100013A	India
16	CR 100484A	India
17	CR 100383	India
18	IRGC 80600	India
19	CR 100004	India
20	CR 100005	India
21	CR 100484	India
22	CR 100490	India
23	IRGC 106150	Laos
24	IRGC 88818	Laos
25	IRGC 106162	Laos
26	IRGC 106156	Laos
27	IRGC 83810	Myanmar
28	IRGC 83811	Myanmar
29	IRGC 83814	Myanmar
30	IRGC 83831	Myanmar
31	IRGC 80762A	Myanmar
32	IRGC 86451	Myanmar
33	IRGC 81989	Myanmar
34	IRGC 93200	Nepal
35	IRGC 93203	Nepal
36	IRGC 93215	Nepal
37	IRGC 93216	Nepal
38	IRGC 93283	Nepal
39	IRGC 93204	Nepal
40	IRGC 93210	Nepal
41	IRGC 93285	Nepal
42	IRGC 93281	Nepal
43	IRGC 81589	PNG
44	IRGC 81996	PNG
45	IRGC 82979	PNG
46	IRGC 82989	PNG
47	IRGC 106506	PNG
48	IRGC 106504	PNG
49	IRGC 100588	Taiwan
50	IRGC 104852	Thailand
51	IRGC 101941	Thailand
52	IRGC 104395	Thailand
53	IRGC 104397	Thailand
54	IRGC 104459	Thailand
55	IRGC 104639	Thailand
56	IRGC 104712	Thailand
57	IRGC 104433	Thailand
58	IRGC 104404A	Thailand
59	IRGC 104404C	Thailand
60	IRGC 104716	Thailand
61	IRGC 106169	Vietnam
62	IRGC 106407	Vietnam
63	IRGC 106413	Vietnam
64	IRGC 113651	Vietnam
65	IRGC 99552	Vietnam
66	IRGC 83819A	Vietnam
67	IRGC83821	Vietnam
68	Vandana	

*Codes: IRGC represents O. rufipogon accessions from the International Rice Genetic Consortium, IRRI, Philippines; CR represents accessions from National Rice Research Institute, Cuttack, India; and PNG represent accession from Papua New Guinea*.

### DNA extraction and SSR marker analysis

Genomic DNA of 182 accessions along with cultivated varieties was isolated using modified Cetyltrimethyl ammonium bromide (CTAB) method of Saghai-Maroof et al. (1984). Eleven SSR markers comprised of 7 dominant SSR markers (*Pup1*-K41, *Pup1*-K42, *Pup1*-K43, *Pup1*-K46, *Pup1*-K48, *Pup1*-K52, and *Pup1*-K59) in INDEL region and 4 co-dominant (*Pup1*-K4, *Pup1*-K5, *Pup1*-K20, and *Pup1*-K29) markers located in *Pup1* genomic region (Chin et al., 2010, 2011) were used for SSRs genotyping (Supplementary Table S2, Supplementary Figure S1). PCR amplification was performed in a 20 ul reaction mix with the following thermal conditions: 94°C for 4 min, followed by 35 cycles of 94°C for 1 min, 55°C for 1 min and 72°C for 1 min and the final extension of 7 min at 72°C.

### Sequencing of *PSTOL1* gene in *O. rufipogon* accessions

The *Oryza sativa* cv. Kasalath sequence (Accession AB458444.1) from position 275,525–276,499 bp covering 975 bp CDS region of *PSTOL1* was used for designing sequencing primers (*PSTOL1* forward: 5′-ATAGCAGGCATTTCTGGCTCA-3′ and *PSTOL1* reverse: 5′-CCATGACAGCTGATTGCCTT-3′). The amplicons were purified using Wizard® SV 96 PCR clean up/Gel extraction kit from Promega, USA, as per manufacturer's protocol. Sequencing reaction was performed using ABI Big-dye Terminator v3.1 chemistry and sequenced using ABI Sequencer 3730XL. Hi-fidelity long-read DNA polymerase (*Phusion Taq*) from Promega, USA, was employed to obtain the required amplicon size. A minimum of three replications was carried out for the confirmation of single nucleotide polymorphism (SNPs).

### Haplotype determination and protein prediction

For comparative sequence analysis, the *PSTOL1* sequences were trimmed to remove any poor quality region at both ends. Multiple sequence alignment was performed using Clustal W of MEGA version 7.0 (Kumar et al., 2016). Kasalath sequence was used as a reference for detection and determination of SNPs position among the *PSTOL1* sequences obtained from *O. rufipogon* accessions. The identified SNPs were manually confirmed using chromatograms. DnaSP version 5.0 and Selecton server (http://selecton.tau.ac.il/, Stern et al., 2007) were used to calculate summary statistics for nucleotide diversity (π), the number of segregating sites, non-synonymous (k_a_), and synonymous (k_s_) mutations and the ratio of k_a_/k_s_ is to estimate positive/purifying selection of a given amino acid, the number of haplotype and Tajima's D test.

Bioinformatics toolkit (http://toolkit.tuebingen.mpg.de/) was used to predict protein structures of all sequences. Homology modeling approach was employed using the Modeler to determine the structure of proteins based on the known structure of template protein. Protein domains were predicted and compared using Pfam (http://pfam.xfam.org/search) and Prosite (http://prosite.expasy.org) online tools. The protein models were checked for the quality using the Ramachandran plot developed using Procheck through PDBsum (http://www.ebi.ac.uk/thornton-srv/databases/pdbsum). The modeled protein structure was visualized and compared in UCSF Chimera (Pettersen et al., 2004). All the structures were superimposed for observing structural variations.

### Phylogenetic analysis

A phylogenetic tree was generated by MEGA7.0 software using the alignment file obtained earlier. The molecular phylogeny was inferred using the Maximum Likelihood method with 1,000 bootstrap (Tamura and Nei, 1993). All positions containing gaps and missing data were eliminated along with other default settings of the software.

### Validation of haplotypes under phosphorus starvation

For functional validation of *PSTOL1* haplotypes toward phosphorus uptake efficiency, eight accessions with seven different haplotypes along with positive control Vandana and negative control PR121 were grown in replicates under low and high phosphorus conditions in the greenhouse facility following the protocol of Gamuyao et al. (2012). High and low P growth conditions were established by maintaining the NaH_2_PO_4_concentration in the hydroponic media as 100 μM and 10 μM, respectively. The eight accessions (CR 100013, CR 10013A-H2, IRGC 104639-H3, IRGC 104712-H4, IRGC 100588-H8, IRGC 105569-H9, IRGC 81989-H11, and IRGC 106506-H17) along with controls were grown in hydroponics for about 2 months. Due to poor germination of accessions representing remaining haplotypes, they were not included in the present study. The seeds were germinated on the wet filter paper, and four seedling replicates per accession were assayed for each phosphorus treatment. After 10 days of germination, seedlings were transferred to the Styrofoam trays suspended in Yoshida growth media (Yoshida et al., 1976). The nutrient media was changed at every third day. Data for the root length, shoot length, final dry root, and shoot weight were taken after 60 days in growth media. Phosphorus content in roots was measured using Inductively Coupled Plasma Spectrophotometer after digestion in a mixture of HNO_3_, HClO_4_, and H_2_SO_4_ (3:1:1) according to the protocol described by Neelam et al. (2011). The morphological data on root and shoot traits under study along with phosphorus content on dry root weight basis was subjected to the statistical analysis. Student's *t*-test was applied for testing the significance of differences among the means of *O. rufipogon* accessions and the controls.

## Results

### Genotyping of *Pup1* locus using SSR markers

The analyzed co-dominant SSR markers were found monomorphic among all the 182 *O*. *rufipogon* accessions and *indica* rice cultivars (PR114, PR121, PR122, PB3, and Vandana) (Supplementary Table S3). For dominant markers (*Pup1*-K41 to K-59), the presence of Vandana alleles was detected in the majority of the *O. rufipogon* accessions as well as in the modern rice cultivars except for marker K-46. Rice cultivars PR114, PR121, and PR122 did not show any amplification for K-46 marker. This indicates the specificity of K-46 marker for the assessment of phosphorus starvation tolerance.

### Haplotype variations in *PSTOL1* gene

**Figure 1:**
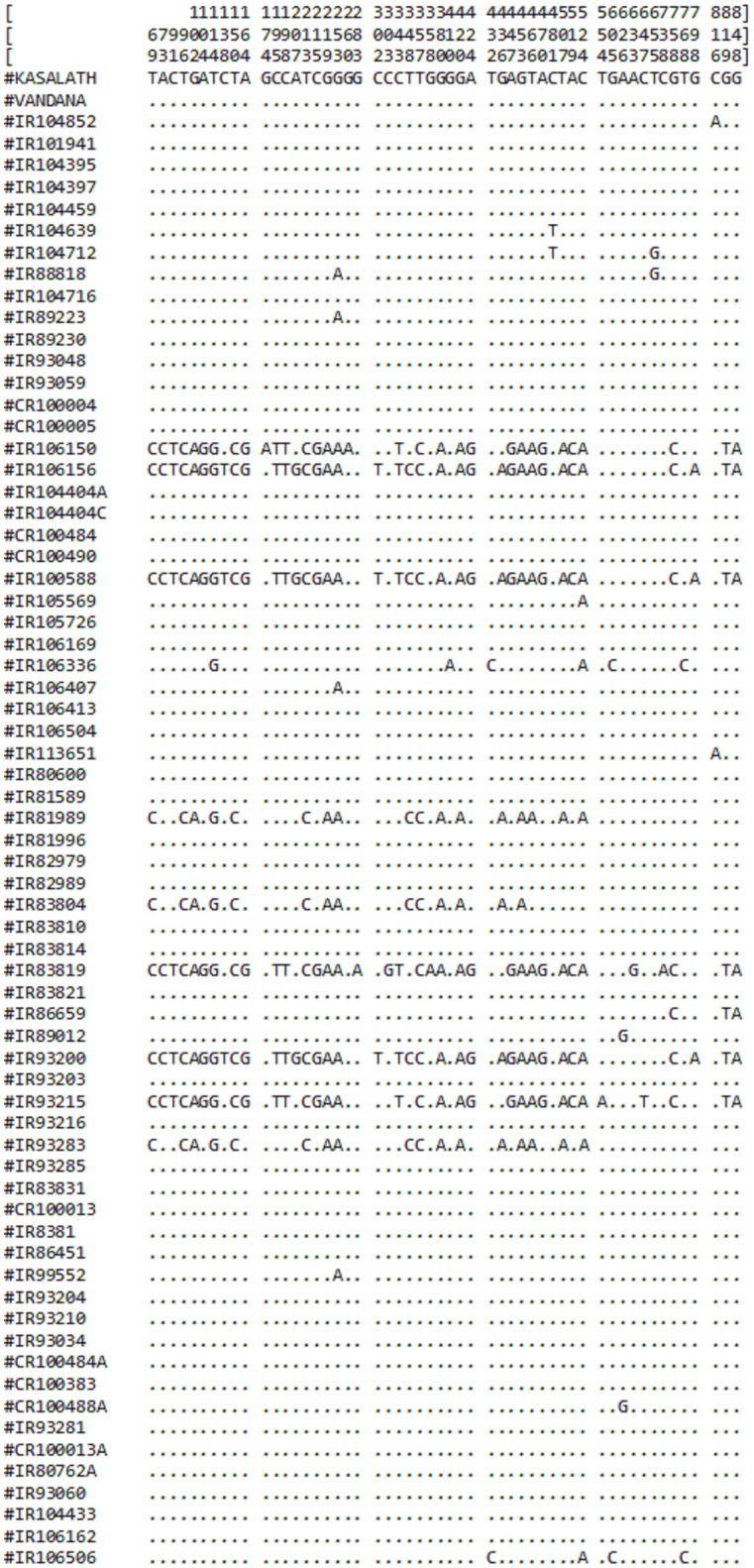
**Schematic representation of identified haplotypes in 67 *O. rufipogon* accessions along with reference sequence Kasalath and Vandana**. Numerical values in vertical lines represent positions of 53 SNPs. The dots (.) represent identical nucleotide at corresponding positions among *O. rufipogon* accessions and the reference sequence.

From comparative sequence analysis, 53 nucleotide changes (52 SNPs and 1 nonsense mutation) across the exon were observed (Table 2). Both types of conversions i.e., transitions (*n* = 39) and transversions (*n* = 14) were observed, while the G/A transition was most common (28.30%). Higher transitions indicated more of the synonymous substitutions were present among genotypes and hence no conformational changes in the structure of proteins were observed. Based on the nucleotide diversity present among *O. rufipogon* accessions, haplotypes were identified using DnaSP software v5.0. Out of 53 identified, 10 SNPs at position 174, 260, 283, 303, 358, 410, 554, 633, 647, and 738 were found as singleton whereas 43 SNPs were parsimony informative sites with a minimum frequency of occurrence in two or more *O*. *rufipogon* accessions (Figure 1). The overall nucleotide diversity (π) of the identified *PSTOL1* alleles was found 0.00758, which indicates low variance in the average number of nucleotide differences per site between two sequences. The number of mutations (*n* = 53) and the number of segregations sites (*S* = 53) were same, suggesting their positive selection. The value of Tajima's D obtained is negative (−1.09788) supporting the above-said statement. Presence of fewer haplotypes was observed than the number of segregating sites indicating the lower frequency of rare alleles present in the population. A total of 17 haplotype groups was formed, revealing genotypes divergence at *PSTOL1* gene among studied *O. rufipogon* accessions (Table 3). The haplotype H1 carried two *O. rufipogon* accessions, one each from Vietnam and Thailand with only one segregating site at position 816. Major haplotype group H2, harbors 44 *O. rufipogon* accessions along with Vandana and Kasalath indicating the sequence similarity among them. The other 16 haplotype groups had *O. rufipogon* accessions ranging from one to three. The haplotype H3 and H4 shares same phylogenetic clade, but having different nucleotide segregating sites. The *O. rufipogon* accessions (IRGC 106150, IRGC 106156, IRGC 93200, IRGC 100588, IRGC 83819, and IRGC 93215) under the smaller phylogenetic clade B grouped into different haplotype i.e., H7, H8, H13, and H16 indicating the presence of rich allelic divergence for the *PSTOL1* gene in these accessions.

**Table 2:** **The total nucleotide variations and post translational modification sites observed at *PSTOL1* among *O. rufipogon* accessions as compared to the reference sequence**.

**S. No**	**Position^#^**	**Alleles**	**Codon change (conservation)**	**Polarity change (Polar/Non Polar)**
1	69	T/C^†^	–	
2	73	A/C^††^	Asn25His (:)	P/P
3	91	C/T^†^	Leu31Phe (:)	NP/NP
4	96	T/C^†^	–	–
5	102	G/A^†^	–	–
6	104	A/G^†^	Lys35Arg (:)	P/P
7	114	T/G^††^	–	–
8	138	C/T^†^	–	–
9	150	T/C^†^	–	–
10	164	A/G^†^	Lys55Arg (:)	P/P
11	174	G/A^†^	–	–
12	195	C/T^†^	–	–
13	198	C/T^†^	–	–
14	207	A/G^†^	–	–
15	213	T/C^†^	–	–
16	215	C/G^††^	Thr72Ser (:)	P/P
17	219	G/A^†^	–	–
18	253	G/A^†^	Gly85Ser (.)	NP/P
19	260	G/A^†^	Ser87Asn (.)	P/P
20	283	G/A^†^	Val95Ile (:)	NP/NP
21	302	C/T^†^	Ser101Phe	P/NP
22	303	C/G^††^	–	–
23	343	C/T^†^	Pro115Ser	P/P
24	348	T/C^†^	–	–
25	357	T/C^†^	Asp120Asn (:)	P/P
26	358	G/A^†^	–	–
27	380	G/A^†^	Ser127Asn (.)	P/P
28	410	G/A^†^	Trp137^*^	NP/^*^
29	420	G/A^†^	–	–
30	424	A/G^†^	Asn142Asp (:)	P/P
31	432	T/C^†^	–	–
32	436	G/A^†^	Gly146Arg	NP/P
33	447	A/G^†^	–	–
34	453	G/A^†^	–	–
35	466	T/A^††^	Cys156Ser (.)	NP/P
36	470	A/G^†^	His157Arg (:)	P/P
37	481	C/T^†^	Arg161Cys	P/NP
38	507	T/A^††^	–	–
39	519	A/C^††^	–	–
40	524	C/A^††^	Ala175Asp	NP/P
41	554	T/A^††^	Phe185Tyr (:)	NP/P
42	605	G/A^†^	Gly202Ala (.)	NP/NP
43	626	A/G^†^	Tyr209Cys	P/NP
44	633	A/G^†^	–	–
45	647	C/T^†^	Ser216Phe	P/NP
46	655	T/G^††^	Tyr219Asp	P/P
47	738	C/A^††^	Asn246Lys (:)	P/P
48	758	G/C^††^	Ser253Thr (:)	P/P
49	768	T/C^†^	–	–
50	798	G/A^†^	–	–
51	816	C/A^††^	–	–
52	819	G/T^††^	Glu273Asp (:)	P/P
53	848	G/A^†^	Arg283Lys (:)	P/P

# *The SNP position was calculated from the translation start site of PSTOL1 gene*.

*Transitions^†^ and transversionsobserved^††^ as nucleotide substitutions*.

* *Stop codon*.

*In parenthesis, conservative mutations were marked as (:), semi-conservative (.), and non-conservative/radical mutations were unmarked*.

**Table 3:** **List of *O. rufipogon* accessions with haplotypes of *PSTOL1* gene**.

**Haplotypes (H)**	***O. rufipogon* accessions**
H1	IRGC 104852, IRGC 113651
H2	Kasalath, Vandana, IRGC 101941, IRGC 104395, IRGC 104397, IRGC 104459, IRGC 104716, IRGC 89230, IRGC 93048, IRGC 93059, CR 100004, CR 100005, IRGC 104404A, IRGC 104404C, CR 100484, CR 100490, IRGC 105726, IRGC 106169, IRGC 106413, IRGC 106504, IRGC 80600, IRGC 81589, IRGC 81996, IRGC 82979, IRGC 82989, IRGC 83810, IRGC 83814, IRGC 83821, IRGC 93203, IRGC 93216, IRGC 93285, IRGC 83831, CR 100013, IRGC 8381, IRGC 86451, IRGC 93204, IRGC 93210, IRGC 93034, CR 100484A, CR 100383, IRGC 93281, CR 100013A, IRGC 80762A, IRGC 93060, IRGC 104433, IRGC 106162
H3	IRGC 104639
H4	IRGC 104712
H5	IRGC 88818
H6	IRGC 89223, IRGC 106407, IRGC 99552
H7	IRGC 106150
H8	IRGC 106156, IRGC 100588, IRGC 93200
H9	IRGC 105569
H10	IRGC 106336
H11	IRGC 81989, IRGC 93283
H12	IRGC 83804
H13	IRGC 83819
H14	IRGC 86659
H15	IRGC 89012, CR 100488A
H16	IRGC 93215
H17	IRGC 106506

*Codes: IRGC represents O. rufipogon accessions from the International Rice Genetic Consortium, IRRI, Philippines; CR represent accessions from National Rice Research Institute, Cuttack, India*.

### Protein structure prediction and comparison

A total of 28 differences in amino acid sequences with a comparison to the variety Vandana and Kasalath were identified (Table 2). The ratio of non-synonymous/synonymous site (k_a_/k_s_) was found 1.52, suggesting that the amino acids were under positive selection and favored by the environment. The amino acids at position 25, 31, 35, 55, 72, 85, 87, 95, 101, 115, 120, 127, 142, 146, 156, 157, 161, 185, 202, 209, 216, 219, 246, 253, 273, and 283 highlighted by yellow color were under positive selection (Supplementary Figure S2).

**Figure 2:**
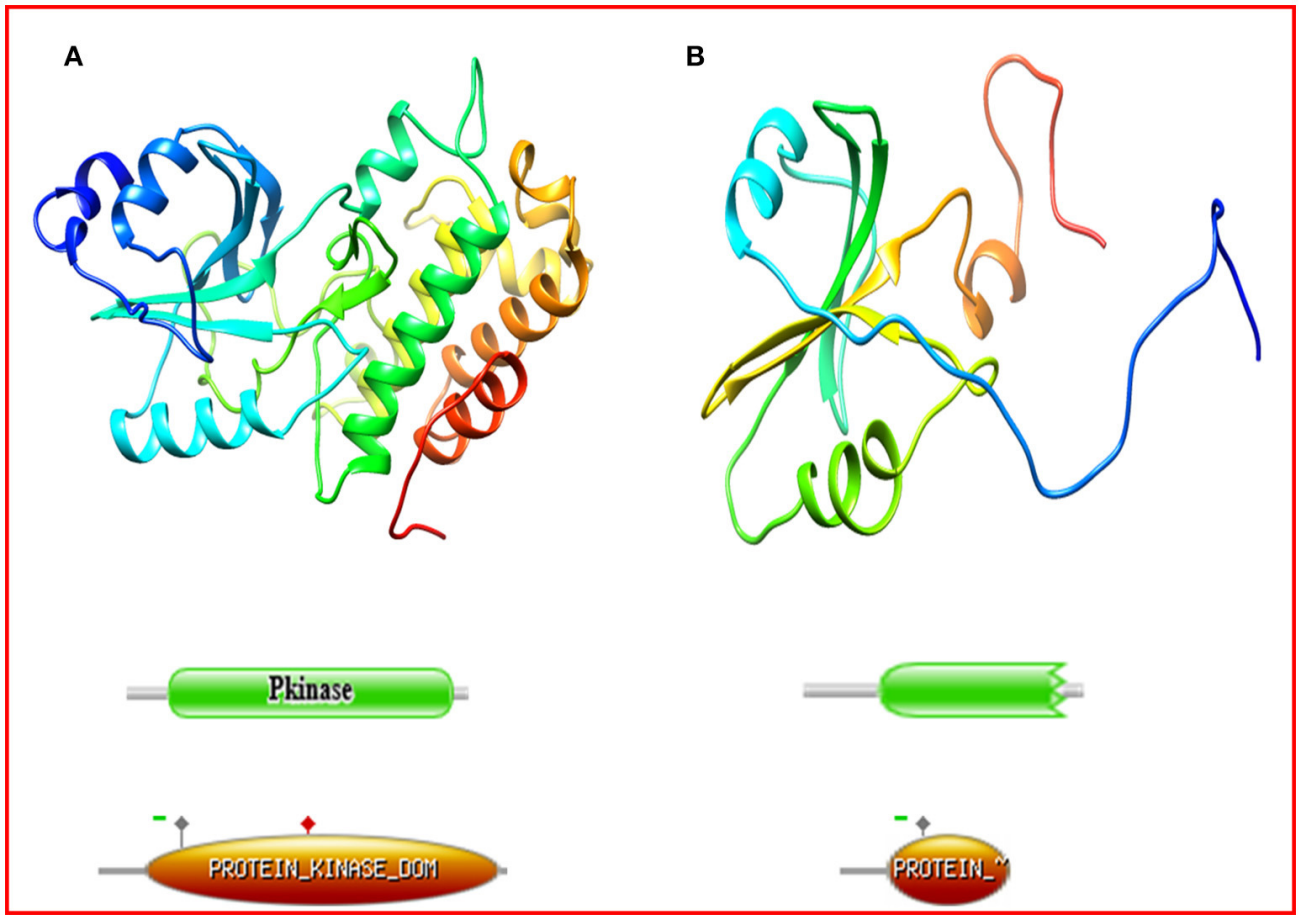
**Comparison of the protein structure of (A)** Kasalath, **(B)** *Oryza rufipogon* accession IRGC 106336. The Kasalath protein model represents leucine rich repeat protein kinase whereas IRGC 106336 showed tyrosine protein kinase domain. As depicted by Prosite results, the encoded structure of protein in Kasalath (green domains by Pfam *E*-value = 5.8e-45; orange domains by Prosite with score = 36.994) showed proton acceptor site (active site, solid red square marked on the orange domain) whereas in *O. rufipogon* accession IRGC 106336 (green domains by Pfam *E*-value = 2.4e-16; orange domains by Prosite score = 14.054) the active site was absent due premature stop codon.

Protein structures for Kasalath and 67 accessions of *O. rufipogon* belonging to different haplotype groups were superimposed and analyzed for structural differences. The non-synonymous mutations concentrate around the ATP binding site (LEU45, ARG47, GLY48, VAL53, ALA65, GLU112, MET114, TYR113, SER118, LYS168, GLN170, and LEU173). (Supplementary Figure S3). The Ramachandran plot of Kasalath revealed more than 99.3% residues were at the core and allowed region and only two residues were present in the disallowed region. Similar results were obtained for protein models of other accessions. All the accessions had a three-dimensional structure similar to the reference Kasalath except accession IRGC 106336 (Figure 2). The protein structure of Kasalath and other accessions displayed 14 helices and two beta-pleated sheets and nine strands, while accession IR 106336 showed only three helices, one sheet, and five strands. In accession IRGC 106336, the *PSTOL1* sequence revealed the presence of premature stop codon at position 137 and its further domain analysis using Pfam and Prosite revealed that it encodes partial protein kinase domain instead of protein tyrosine kinase as encoded by Kasalath. The Prosite analysis for *PSTOL1* protein in Kasalath predicted the features as the kinase domain from codon 39–319, nucleotide phosphate binding site (NP_BIND) at position 45–53, ATP-binding site (BINDING) at position 67 and the proton acceptor site as active site at position 166 whereas IRGC 106336 had partial protein kinase domain from 39 to 136, NP_BIND from position 45–53, ATP-binding site (BINDING) at position 67, with absence of proton acceptor site i.e., active site (Supplementary Table S4).

### Phylogenetic analysis

**Figure 3:**
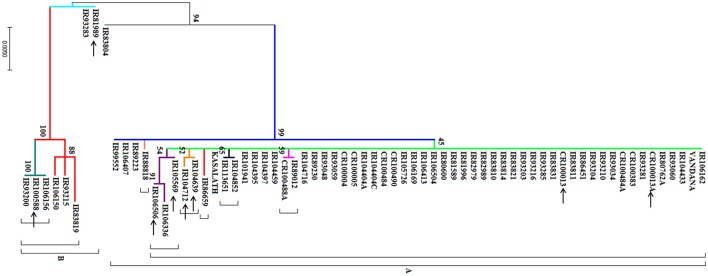
**The evolutionary history was inferred by using the Maximum Likelihood method based on the Tamura-Nei model**. The bootstrap consensus tree inferred from 1,000 replicates is taken to represent the evolutionary history of the taxa analyzed. Branches corresponding to partitions reproduced in less than 50% bootstrap replicates are collapsed. Initial tree(s) for the heuristic search were obtained automatically by applying Neighbor-Join and BioNJ algorithms to a matrix of pairwise distances estimated using the Maximum Composite Likelihood (MCL) approach, and then selecting the topology with superior log likelihood value. The different node colors indicate the presence of different mutations as compared to the reference. The *O. rufipogon* accessions used for validation under phosphorus deficiency are indicated by an arrow.

Phylogenetic analysis at *PSTOL1* locus revealed divergence among *O. rufipogon* accessions (Figure 3). The different node colors correspond to the different mutations present in *O. rufipogon* accessions and vice-versa. Two major groups were observed. The clade A is consisted of 59 *O*. *rufipogon* accessions which can be further divided into 8 subgroups. Out of 59 accessions, 44 were found to have similar sequences as that of the reference sequence, whereas others having either single or more substitutions as compared to the reference. Among 44 accessions 6 are of Nepal origin, 6 are of Cambodia origin, 9 are of Indian origin, 8 are of Thailand origin, 4 are of Papua New Guinea origin, 4 are of Vietnam origin, 1 is of Laos origin, and 6 are of Myanmar origin. This indicates a common evolutionary relationship of these *O. rufipogon* accessions with aus type variety Kasalath. The clade B includes eight accessions, two from Laos (IRGC 106150, IRGC 106156), three from Nepal (IRGC 93200, IRGC 93215, IRGC 93283) and one each from Vietnam (IRGC 83819), Taiwan (IRGC 100588), Myanmar (IRGC 81989).

### Validation of novel alleles under phosphorus deficiency

Genotypic variation for root and shoot length, dry root and shoot weight and phosphorus content on dry roots basis were examined under phosphorus sufficient and deficient conditions after 2 months of the experiment (Tables 4A,B). Genotypic differences were observed among all *O*. *rufipogon* accessions under both growing conditions. All of the genotypes under phosphorus sufficient conditions had almost double root length as compared to the deficient conditions. Though, not much difference was observed in shoot length and shoot weight for all the genotypes when compared under phosphorus sufficiency and deficiency conditions. The *O. rufipogon* accession IRGC 106506 (H17) showed the best root and shoot length under phosphorus-deficient conditions when compared to other genotypes and control. Among H2 haplotype *O. rufipogon* accession CR 100013A performed better than Vandana for all the traits studied. In terms of root and shoot weight, *O. rufipogon* IGCC 106506 (H17) showed the best root weight followed by IRGC 81989 from H11 haplotype. Approximately, 1.5 and 2.3 times higher phosphorus content was found in the *O. rufipogon* accession IRGC 106506 when compared to *indica* cultivar PR 121 and Vandana respectively. Other than that, *O. rufipogon* accession IRGC 104639 from H3 haplotype also showed 1.2 times higher phosphorus content when compared to PR121 indicating their potentiality toward improving elite cultivars for phosphorus uptake.

**Table 4:** **Morphological data on root and shoot length, root and shoot weight and phosphorus content under phosphorus sufficient conditions (A), and phosphorus deficiency conditions (B)**.

**(A) PHOSPHORUS SUFFICIENT CONDITIONS**						
**Genotypes**	**Haplotype**	**Root length (cm)**	**Shoot length (cm)**	**Root weight (g)**	**Shoot weight (g)**	**Phosphorus content on dry root basis (ppm)**
CR 100013	H2	16.11^f^ ± 1.29	101.5^ef^ ± 12.02	0.08^a^ ± 0.01	0.59^a^ ± 0.12	11003.72^a^ ± 521.60
CR 100013A	H2	19.39^i^ ± 1.32	107.29^f^ ± 5.36	0.16^c^ ± 0.02	0.98^cd^ ± 0.04	11886.22^a^ ± 564.09
IRGC 104639	H3	17.25^g^ ± 2.12	93.09^e^ ± 3.75	0.15^c^ ± 0.01	0.87^c^ ± 0.06	18385.15^g^ ± 590.72
IRGC 104712	H4	14.98^d^ ± 1.63	70.63^c^ ± 2.64	0.14^bc^ ± 0.03	0.69^b^ ± 0.04	20106.73^hi^ ± 904.54
IRGC 100588	H8	11.13^b^ ± 0.83	83.40^d^ ± 4.46	0.08^a^ ± 0.02	0.56^a^ ± 0.18	15749.05^e^ ± 570.48
IRGC 105569	H9	18.50^h^ ± 1.41	117.04^g^ ± 3.24	0.12^b^ ± 0.03	1.05^d^ ± 0.01	19869.51^h^ ± 982.04
IRGC 81989	H11	17.58^g^ ± 2.44	98.31^e^ ± 2.91	0.15^c^ ± 0.02	0.97^cd^ ± 0.04	19217.18^h^ ± 312.17
IRGC 106506	H17	17.31^g^ ± 1.94	106.5^f^ ± 4.94	0.18^d^ ± 0.01	0.89^c^ ± 0.08	18712.37^g^ ± 471.31
PR121	–	16.03^f^ ± 1.39	52.635^a^ ± 2.97	0.12^b^ ± 0.02	0.66^a^ ± 0.09	19927.63^h^ ± 277.44
Vandana	H2	10.43^a^ ± 0.64	81.35^d^ ± 1.27	0.08^a^ ± 0.01	0.51^a^ ± 0.06	14144.01^e^ ± 347.39
**(B) PHOSPHORUS DEFICIENCY CONDITIONS**						
CR100013	H2	6.00^a^ ± 1.50	79.25^bc^ ± 11.75	0.041^a^ ± 0.01	0.628^a^ ± 0.11	4077.15^d^ ± 180.35
CR10013A	H2	7.00^b^ ± 1.75	92.87^c^ ± 14.37	0.082^b^ ± 0.02	1.066^d^ ± 0.04	7352.75^fg^ ± 507.25
IRGC104639	H3	6.87^b^ ± 0.12	94.75^c^ ± 4.75	0.111^bc^ ± 0.02	0.847^c^ ± 0.01	8931.65^i^ ± 423.34
IRGC104712	H4	6.87^b^ ± 0.37	81.37^bc^ ± 2.37	0.137^c^ ± 0.02	0.726^b^ ± 0.05	7710.05^g^ ± 260.05
IRGC100588	H8	4.37^a^ ± 0.87	105.41^d^ ± 7.08	0.021^a^ ± 0.02	0.542^a^ ± 0.10	2147.55^a^ ± 326.95
IRGC105569	H9	7.75^b^ ± 1.00	105^d^ ± 9.25	0.094^bc^ ± 0.02	1.083^d^ ± 0.03	7105.05^g^ ± 739.95
IRGC81989	H11	7.62^b^ ± 0.37	93.62^c^ ± 7.62	0.137^c^ ± 0.01	1.128^d^ ± 0.12	6575.05^f^ ± 159.96
IRGC106506	H17	8.75^c^ ± 0.25	111.12^d^ ± 8.37	0.158^d^ ± 0.03	0.976^d^ ± 0.14	10015.00^j^ ± 60.0
PR121	–	6.87^b^ ± 0.37	51.37^a^ ± 1.12	0.098^b^ ± 0.01	0.569^a^ ± 0.04	6955.00^f^ ± 485.0
Vandana	H2	5.04^a^ ± 0.29	88.75^c^ ± 0.50	0.056^a^ ± 0.01	0.665^a^ ± 0.11	4418.75^d^ ± 471.75

*Superscripts (a–j) represents significant differences in the means of different O. rufipogon accessions and control based on t-test, at p < 0.05*.

## Discussion

### Allelic differences among *O. rufipogon* accessions and *O. sativa* with *Pup1* specific markers

Our results with *Pup1* specific markers on *O. rufipogon* accessions and *O. sativa* cultivars revealed no allelic differences for almost all markers other than K-46 and K-05 (Supplementary Table S3). Chin et al. (2010), also observed the Kasalath specific alleles for markers K-41, K-43, and K-48 in lowland/irrigated (*indica, japonica, aus*, and traditional or modern) rice cultivars, representing nonusefulness of these markers for marker aided selection for phosphorus uptake efficiency. Similarly, the markers K-42, and K-29 were not found linked with PUE by Sarkar et al. (2011) while assessing *indica* germplasm. It should be taken into consideration that Gamuyao et al. (2012) ruled out other co-dominant and INDEL markers as indicative of PUE except for K-46. This dominant marker was found useful for MAS in the progenies involving Kasalath as *Pup1* donor variety and Asian lowland rice varieties (without this gene) by Chin et al. (2010, 2011) and Pariasca-Tanaka et al. (2014), supporting our results. Mukherjee et al. (2014), assessed 108 genotypes from different states of India for phosphorus acquisition efficiency with gene specific markers and closely linked SSR marker RM1261 and reported no association between markers and PUE. The same has been observed when they studied a RIL population developed from a cross between Gobindabhog (with *PSTOL1* gene) and Satabdi (*PSTOL1* absent). The notion that *PSTOL1* specific marker was not indicative in the case of *indica* germplasm (Mukherjee et al., 2014) is more likely due to the complex nature of *Pup1* locus and different genetic background and environment where this gene has to express.

The germplasm survey with *Pup1* specific markers of Kasalath indicated entire inserted region of 90 kb among studied *O. rufipogon* accessions. The probable explanation for this could be a continuous gene flow between *O*. *sativa* and *O. rufipogon* populations throughout the history of domestication (Vaughan et al., 2008). Also, *O. rufipogon* accessions from South and Southeast Asia are considered as the wild progenitor of domesticated rice (Oka, 1988; Molina et al., 2011) and hence chances of recent hybridization events needs to be accounted for the phenomena. While studying allelic diversity at *PSTOL1*, Vigueira et al. (2016) reported presence/absence polymorphism in 12 of the *O. rufipogon* accessions out of 40 studied, along with the loss of function mutation in one accession and 56 synonymous and nonsynonymous substitutions in 28. He explains this phenomenon as long- term balancing selection at *PSTOL1* locus for maintaining both functional and non-functional alleles among the accessions of *O. rufipogon* and *indica, aus, tropical japonica* cultivars. Though, none of the alleles found conferring superior phenotype than Kasalath in their study, whereas in our case functional alleles were observed.

### Phylogeography of *O. rufipogon* accessions under study

Our results on molecular diversity at *PSTOL1* locus, suggests the presence of lower diversity among *O. rufipogon* accessions from South Asia and Southeast Asian nations. This is expected as they share common geographical boundaries. This result is in accordance with several studies conducted on the assessment of genetic diversity of Asian wild rice using RFLP, microsatellite markers, SINEs, sequence based polymorphism, ISSRs, chloroplast, and low-copy nuclear markers (Joshi et al., 2000; Cheng et al., 2002; Rakshit et al., 2007; Xu et al., 2007; Huang et al., 2012). In a study, conducted by Huang et al. (2012) on the phylogeography of Asian wild rice using 42 genome-wide sequence tagged sites demonstrated that *O. rufipogon* accessions were grouped into two genetically distinct clades (Ruf-I and Ruf-II). The *O. rufipogon* accessions from South Asia and Indochinese Peninsula (Thailand, Myanmar, Cambodia, Vietnam, and Laos), were clustered into one group (Ruf-II), supporting our results. The presence of few accessions from Nepal, Laos, Vietnam, Cambodia, and Myanmar into intermediate and second major clade may be interpreted as an admixture. Moreover, the humid tropical plain areas in the Indo Peninsula zone act as a transitional region for evolutionary studies is likely the reasons for observed admixture.

### Haplotype diversity at *PSTOL1* and contribution toward phosphorus uptake efficiency

It is always worthwhile to look for better alleles of a gene for creating and maintaining natural genetic diversity. Our results demonstrated the presence of 17 different haplotypes within 975 bp sequence of *PSTOL1* locus indicating rich nucleotide variation among studied *O*. *rufipogon* accessions. Two of the *O. rufipogon* accessions under H17 and H11 were found performing better than the positive control under phosphorus-deficient conditions. Functional allelic variants were observed and utilized for improving various agronomically important traits in cereal crops (Ellis and Setter, 1999; Bhullar et al., 2010; Ravensdale et al., 2012; Vasudevan et al., 2014; Ashkani et al., 2015). The wheat powdery mildew resistance gene *Pm3* with 17 identified functional alleles is a remarkable example of natural variations present in GenBank accessions and can be efficiently utilized for conferring broad-spectrum disease resistance. For rice blast resistance gene, functional orthologous have been found in wild rice *O. rufipogon* accessions (Lv et al., 2013; Xu et al., 2014; Ashkani et al., 2015) defining their utility in widening the genetic base of cultivated rice varieties. A novel allele of *PSTOL1* gene is identified in *O. glaberrima* (CG14) and being transferred to the NERICAs (New Rice for Africa) cultivars using allele-specific markers. In their study, they identified 3 novel alleles in 10 studied *O. rufipogon* accessions and also the presence of Kasalath alleles for INDEL markers which is consistent with our results. The successful efforts for the transfer of *PSTOL1* were made by Gamuyao et al. (2012) through marker assisted backcross breeding to Asian rice cultivar IR74 with increased root growth and phosphorus uptake efficiency.

The presence of *PSTOL1* in all *O. rufipogon* accessions raise the question regarding the functionality of different alleles under phosphorus deficiency. Validation of haplotype groups showed the significant difference for root and shoot length and biomass as compared to PR121 and Vandana under both phosphorus sufficient and deficient conditions. The correlation between root elongation, higher root and shoot biomass of genotypes under P-deficiency is considered as one of an important indicator of higher phosphorus uptake efficiency. A number of reports, including QTLs on P deficiency induced root elongation in plants were published (Steingrobe et al., 2001; He et al., 2003; Ma et al., 2003; Wissuwa, 2005; Li et al., 2007; Rose et al., 2013). Near isogenic line of “Nipponbare” with *Pup1* QTL from “Kasalath” showed high P content, high tillering and high root growth under P-deficient upland conditions (Wissuwa and Ae, 2001; Wissuwa et al., 2002). The *O. rufipogon* accession IRGC 106506 showed the highest root growth under P-deficiency and thus is the best option for transferring this novel allele to elite cultivars for improving P starvation tolerance.

## Conclusion

In Summary, our efforts for harnessing superior allele of *PSTOL1* in *O. rufipogon* revealed three accessions (IRGC 106506, IRGC 81989, and IRGC 104639) from haplotypes H17, H11, and H3 with better performance under Phosphorus deficiency conditions. Though, further confirmation of identified superior alleles should be done under the phosphorus-deficient soil. Transfer and development of allele-specific markers for MAS have already been initiated at Punjab Agricultural University. Marker assisted transfer of these potential haplotypes to the *indica* rice cultivars would be useful to breed better rice with sustainable yield under phosphorus-deficient soil.

## Author contributions

Conceived and designed the experiment: KN, KS, and SD. Performed the experiment: KN, ST, N, and KK. Analyzed the data: KN, ST, and IY. Wrote the paper: KN and ST.

### Conflict of interest statement

The authors declare that the research was conducted in the absence of any commercial or financial relationships that could be construed as a potential conflict of interest.

## Supplementary material

Supplementary Figure S1 ***Pup1*** **genomic region with positions of co-dominant and dominant markers**.

Supplementary Figure S2 **Evolutionary sweeps or selections of protein sequences at *PSTOL1* gene: Positive selection is colored in shades of yellow, and purifying selection is colored in shades of magenta**.

Supplementary Figure S3 **Superimposed protein model of *PSTOL1* gene of all *O. rufipogon* accessions using UCSF Chimera**. Ball and sticks represent the mutated residues of haplotypes.

Supplementary Table S1 **List of *Oryza rufipogon* accessions selected for SSR marker analysis along with countries of origin**.

Supplementary Table S2 **SSR primers used to study variability among *O. rufipogon* accessions**.

Supplementary Table S3 **Genotyping results of *O. rufipogon* accessions using eleven SSR markers of *Pup 1* locus**.

Supplementary Table S4 **The Prosite analysis of *PSTOL1* protein model of Kasalath and *Oryza rufipogon* accession IRGC 106336**.
