# Supplementary figures and images for: Novel Alleles of Phosphorus-Starvation Tolerance 1 Gene (PSTOL1) from Oryza rufipogon Confers High Phosphorus Uptake Efficiency
Source: Front Plant Sci. 2017 Apr 11;8:509. doi: 10.3389/fpls.2017.00509 (PMC5387083; doi:10.3389/fpls.2017.00509)

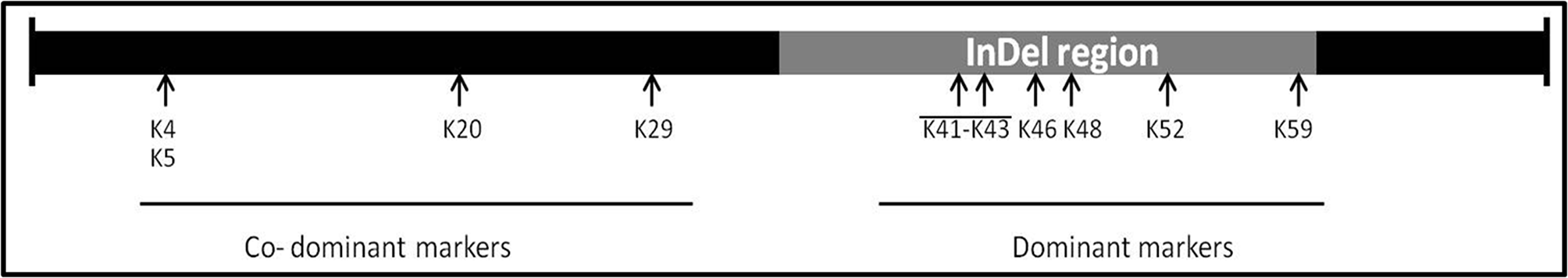

Supplement: Supplementary Figure S1 — Pup1 genomic region with positions of co-dominant and dominant markers. [file Image1.TIF]

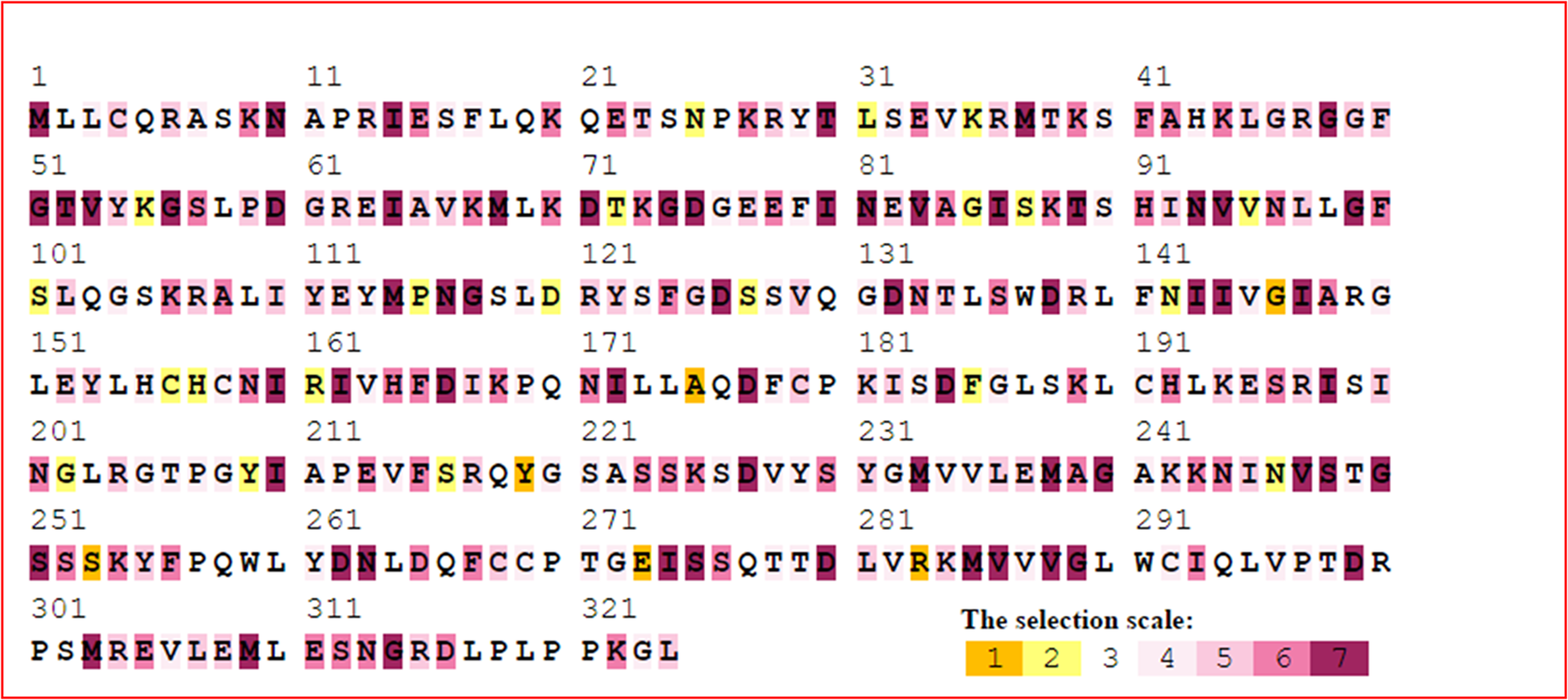

Supplement: Supplementary Figure S2 — Evolutionary sweeps or selections of protein sequences at PSTOL1 gene: Positive selection is colored in shades of yellow, and purifying selection is colored in shades of magenta. [file Image2.TIF]

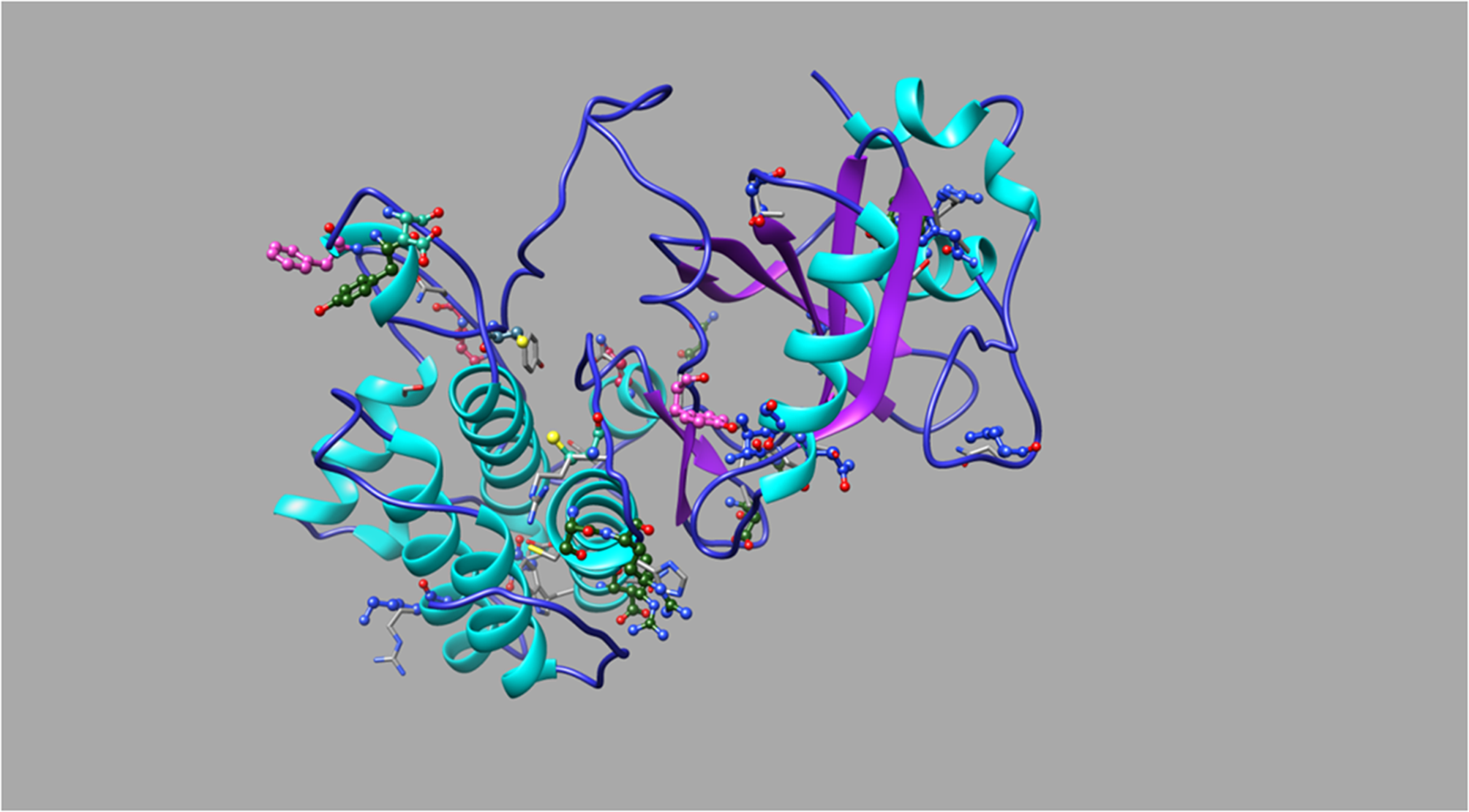

Supplement: Supplementary Figure S3 — Superimposed protein model of PSTOL1 gene of all O. rufipogon accessions using UCSF Chimera. Ball and sticks represent the mutated residues of haplotypes. [file Image3.TIF]
