# Supplementary material for: Novel Alleles of Phosphorus-Starvation Tolerance 1 Gene (PSTOL1) from Oryza rufipogon Confers High Phosphorus Uptake Efficiency
Source: Front Plant Sci. 2017 Apr 11;8:509. doi: 10.3389/fpls.2017.00509 (PMC5387083; doi:10.3389/fpls.2017.00509)
Supplement: Supplementary Table S1 — List of Oryza rufipogon accessions selected for SSR marker analysis along with countries of origin. [file Table1.PDF]

Supplementary Table S1: List of *Oryza rufipogon* accessions selected for SSR marker analysis along with countries of origin.

S. No.	Accession Number	Origin
1	IR104389	Bangladesh
2	IR105883	Bangladesh
3	IR105884	Bangladesh
4	IR105887	Bangladesh
5	IR88783	Bangladesh
6	IR88784	Bangladesh
7	IR88788	Bangladesh
8	IR88789	Bangladesh
9	IR105569	Cambodia
10	IR105726	Cambodia
11	IR105735	Cambodia
12	IR106326	Cambodia
13	IR106336	Cambodia
14	IR110406	Cambodia
15	IR81986	Cambodia
16	IR83804	Cambodia
17	IR86549	Cambodia
18	IR86555	Cambodia
19	IR86556	Cambodia
20	IR86659	Cambodia
21	IR89006	Cambodia
22	IR89008	Cambodia
23	IR89012	Cambodia
24	IR89013	Cambodia
25	IR89020	Cambodia
26	IR89046	Cambodia
27	IR89223	Cambodia
28	IR89224	Cambodia
29	IR89230	Cambodia
30	IR93014	Cambodia
31	IR93034	Cambodia
32	IR93043	Cambodia
33	IR93048	Cambodia
34	IR93058	Cambodia
35	IR93059	Cambodia

S. No.	Accession Number	Origin
36	IR93060	Cambodia
37	IR93062	Cambodia
38	IR93063	Cambodia
39	IR96962	Cambodia
40	CR100001	India
41	CR100004	India
42	CR100005	India
43	CR100005B	India
44	CR100006	India
45	CR100006A	India
46	CR100007	India
47	CR100013	India
48	CR100013A	India
49	CR100015	India
50	CR100018	India
51	CR100029	India
52	CR100035A	India
53	CR100055	India
54	CR10018A	India
55	CR10018A	India
56	CR100375	India
57	CR100379A	India
58	CR100381	India
59	CR100383	India
60	CR100401	India
61	CR100402	India
62	CR100484	India
63	CR100484A	India
64	CR100488A	India
65	IR106081	India
66	IR80433	India
67	IR80433	India
68	IR80433A	India
69	IR80433B	India
70	IR80562	India

S. No.	Accession Number	Origin
71	IR80600	India
72	IR80600	India
73	IR80610	India
74	IR80660	India
75	IR81885	India
76	IR81885	India
77	IR93137	India
78	IR106149	Laos
79	IR106150	Laos
80	IR106152	Laos
81	IR106156	Laos
82	IR106161	Laos
83	IR106162	Laos
84	IR88818	Laos
85	IR99549	Laos
86	IR100923	Myanmar
87	IR105494	Myanmar
88	IR106343	Myanmar
89	IR106349	Myanmar
90	IR80762	Myanmar
91	IR80762	Myanmar
92	IR80762A	Myanmar
93	IR80762B	Myanmar
94	IR81989	Myanmar
95	IR81991	Myanmar
96	IR83810	Myanmar
97	IR83811	Myanmar
98	IR83813	Myanmar
99	IR83814	Myanmar
100	IR83831	Myanmar
101	IR86451	Myanmar
102	IR105696	Nepal
103	IR93200	Nepal
104	IR93202	Nepal
105	IR93203	Nepal

S. No.	Accession Number	Origin
106	IR93204	Nepal
107	IR93205	Nepal
108	IR93209	Nepal
109	IR93210	Nepal
110	IR93215	Nepal
111	IR93216	Nepal
112	IR93217	Nepal
113	IR93217	Nepal
114	IR93219	Nepal
115	IR93221	Nepal
116	IR93222	Nepal
117	IR93278	Nepal
118	IR93280	Nepal
119	IR93281	Nepal
120	IR93283	Nepal
121	IR93285	Nepal
122	IR 106267	PNG
123	IR 106268	PNG
124	IR 106290	PNG
125	IR 81996	PNG
126	IR106290	PNG
127	IR106503	PNG
128	IR106504	PNG
129	IR106506	PNG
130	IR81589	PNG
131	IR81985	PNG
132	IR81996	PNG
133	IR82979	PNG

S. No.	Accession Number	Origin
134	IR82989	PNG
135	IR100588	Taiwan
136	IR100657	Taiwan
137	IR100678	Taiwan
138	IR103308	Taiwan
139	IR101941	Thailand
140	IR103850	Thailand
141	IR104395	Thailand
142	IR104397	Thailand
143	IR104404	Thailand
144	IR104404A	Thailand
145	IR104404B	Thailand
146	IR104404C	Thailand
147	IR104404D	Thailand
148	IR104404E	Thailand
149	IR104423	Thailand
150	IR104424	Thailand
151	IR104425	Thailand
152	IR104433	Thailand
153	IR104459	Thailand
154	IR104495	Thailand
155	IR104639	Thailand
156	IR104641	Thailand
157	IR104661	Thailand
158	IR104712	Thailand
159	IR104714	Thailand
160	IR104716	Thailand
161	IR104852	Thailand

S. No.	Accession Number	Origin
162	IR81969	Thailand
163	IR106169	Vietnam
164	IR106407	Vietnam
165	IR106413	Vietnam
166	IR106416	Vietnam
167	IR106421	Vietnam
168	IR106422	Vietnam
169	IR106423	Vietnam
170	IR106423	Vietnam
171	IR106424	Vietnam
172	IR106425	Vietnam
173	IR106427	Vietnam
174	IR106427	Vietnam
175	IR106761	Vietnam
176	IR113651	Vietnam
177	IR83819	Vietnam
178	IR83821	Vietnam
179	IR83823	Vietnam
180	IR83824	Vietnam
181	IR99551	Vietnam
182	IR99552	Vietnam
183	Cultivar PR114	India
184	Cultivar PR121	India
185	Cultivar PB3	India
186	Cultivar VANDANA	India

PNG stands for Papua New Guinea. IR numbers are accession numbers from International rice genetic consortium, IRRI, Philippines and CR numbers are accession numbers of NRRI-ICAR, Cuttack, India.
