# Supplementary material for: Novel Alleles of Phosphorus-Starvation Tolerance 1 Gene (PSTOL1) from Oryza rufipogon Confers High Phosphorus Uptake Efficiency
Source: Front Plant Sci. 2017 Apr 11;8:509. doi: 10.3389/fpls.2017.00509 (PMC5387083; doi:10.3389/fpls.2017.00509)
Supplement: Supplementary Table S2 — SSR primers used to study variability among O. rufipogon accessions. [file Table2.PDF]

Supplementary Table S2: SSR primers used to study variability among *O. rufipogon* accessions.

S.No.	Primer*	Primer-pair sequence	Amplicons (bp)
1	*Pup1*-K4	F: 5'-GGGATATCAAGCTTGTGGTG-3'<br>R: 5'-GAATGCTGTTTCGCTTATGG-3'	650
2	*Pup1*-K5	F: 5'-AGTACAGTCCGGCGTCATAC-3'<br>R: 5'-CCGAGATCTGGTCCTCAATA-3'	280
3	*Pup1*-K20	F: 5'-CTGGACTTGACCCCAATGTA-3'<br>R: 5'-TCTGATGGAGTGTTCGGAGT-3'	240
4	*Pup1*-K29	F: 5'-CCAATGCATCCAATTCTTGT-3'<br>R: 5'-ATGAGCCCAGATTACGAATG-3'	480
5	*Pup1*-K41	F: 5'-TGATGAATCCATAGGACAGCGT-3'<br>R: 5'-TCAGGTGGTGCTTCGTTGGTA-3'	382
6	*Pup1*-K42	F: 5'-CCCGAGAGTTCATCAGAAGGA-3'<br>R: 5'-AGTGAGTGGCGTTTGCGAT-3'	918
7	*Pup1*-K43	F: 5'-AGGAGGATGAGCCTGAAGAGA-3'<br>R: 5'-TCGCACTAACAGCAGCAGATT-3'	912
8	*Pup1*-K46	F: 5'-TGAGATAGCCGTCAAGATGCT-3'<br>R: 5'-AAGGACCACCATTCCATAGC-3'	523
9	*Pup1*-K48	F: 5'-CAGCATTCAGCAAGACAACAG-3'<br>R: 5'-ATCCGTGTGGAGCAACTCATC-3'	847
10	*Pup1*-K52	F: 5'-ACCGTTCCCAACAGATTCCAT-3'<br>R: 5'-CCCGTAATAGCAACAACCCAA-3'	505
11	*Pup1*-K59	F: 5'-GGACACGGATTCAAGGAGGA-3'<br>R: 5'-TGCTTTCCATTTGCGGCTC-3'	550

*Chin et al., 2010 and 2011.
