# Supplementary material for: Novel Alleles of Phosphorus-Starvation Tolerance 1 Gene (PSTOL1) from Oryza rufipogon Confers High Phosphorus Uptake Efficiency
Source: Front Plant Sci. 2017 Apr 11;8:509. doi: 10.3389/fpls.2017.00509 (PMC5387083; doi:10.3389/fpls.2017.00509)
Supplement: Supplementary Table S3 — Genotyping results of O. rufipogon accessions using eleven SSR markers of Pup 1 locus. [file Table3.PDF]

Supplementary Table 3: Genotyping results of *O. rufipogon* accessions using eleven SSR markers of *Pup 1* locus.

S. No.	Accession	***Pup*K-04**	****Pup* K-05a**	***Pup*K -05b**	***Pup*K -20**	***Pup*K -29**	**K-41**	**K-42**	**K-43**	**K-46**	**K-48**	**K-52**	**K-59**
1	IR81589	*+	+	-	na	+	+	+	+	+	+	+	+
2	IR99552	na	+	-	na	+	+	+	+	+	+	+	+
3	IR106427	na	+	-	+	+	+	+	+	+	na	+	+
4	IR93200	+	+	-	na	+	+	+	+	+	+	+	+
5	IR93204	+	+	-	+	+	+	+	+	+	+	+	+
6	IR104389	+	+	-	+	+	+	+	+	+	+	+	+
7	IR80762	+	+	-	+	+	+	+	+	+	+	+	+
8	IR81986	+	+	-	+	+	+	+	+	+	+	+	+
9	IR89006	na	+	-	+	+	+	+	+	+	na	+	+
10	IR80433	na	+	-	+	+	+	+	+	+	+	+	+
11	IR81885	+	+	-	+	+	+	+	+	+	+	+	+
12	CR100484	+	na	-	na	+	+	na	+	+	+	+	+
13	IR81996	+	+	-	na	+	+	+	+	+	+	+	+
14	IR83819	+	+	-	+	+	+	+	+	+	+	+	+
15	IR106413	na	+	-	+	+	+	+	+	+	+	+	+
16	IR93278	+	+	-	+	+	+	+	+	+	+	+	+
17	IR93205	+	+	-	+	+	na	+	+	+	na	+	+
18	IR88783	+	+	-	+	+	+	+	+	+	na	+	+
19	IR81991	+	+	-	+	+	+	+	+	+	+	+	+
20	IR83804	+	+	-	+	+	+	+	+	+	+	+	+
21	IR89008	+	+	-	+	+	+	+	+	+	na	+	+
22	IR80433A	+	+	-	+	+	+	na	+	+	+	+	+
23	IR81885	+	+	-	+	+	+	+	+	+	+	+	+
24	CR100484A	+	+	-	+	+	+	+	+	+	+	+	+
25	IR81985	+	+	-	+	+	+	+	+	+	+	+	+
26	IR83821	+	+	-	+	+	+	+	+	+	+	+	+
27	IR106416	na	na	-	+	+	na	+	+	+	+	+	+
28	IR93280	+	+	-	+	+	+	+	+	+	na	+	+
29	IR93209	+	+	-	+	+	+	+	+	+	na	+	+

S. No.	Accession	*Pup*K-04	***Pup* K-05a	*Pup*K -05b	*Pup*K -20	*Pup*K -29	K-41	K-42	K-43	K-46	K-48	K-52	K-59
30	IR88784	+	+	-	+	+	+	+	+	+	+	+	+
31	IR83831	na	+	-	+	+	+	+	+	+	na	+	+
32	IR96962	+	+	-	+	+	+	+	+	+	+	+	+
33	IR105569	+	+	-	+	+	na	+	+	+	+	+	+
34	IR80433B	+	+	-	+	+	+	+	+	+	na	+	+
35	IR106081	+	+	-	+	+	na	+	+	+	+	+	+
36	CR100488A	+	na	-	+	+	+	+	+	+	na	+	+
37	IR 81996	+	+	-	+	+	+	+	+	+	+	+	+
38	IR83823	+	+	-	+	+	na	+	+	+	na	+	+
39	IR106421	+	+	-	+	+	na	+	+	+	+	+	+
40	IR93281	+	+	-	+	+	+	+	+	+	+	+	+
41	IR93210	+	+	-	+	+	+	+	+	+	na	+	+
42	IR88788	+	+	-	+	+	+	+	+	+	+	+	+
43	IR86451	+	+	-	+	+	+	na	+	+	+	+	+
44	IR 106267	+	+	-	+	+	+	+	+	+	+	+	+
45	IR105726	+	+	-	+	+	+	+	+	+	+	+	+
46	IR80562	+	+	-	+	+	+	+	+	+	+	+	+
47	CR100001	+	+	-	+	+	+	+	+	+	+	+	+
48	IR 106268	+	+	-	+	+	+	+	+	+	+	+	+
49	IR83824	+	+	-	+	+	+	+	+	+	+	+	+
50	IR106422	+	+	-	+	+	+	+	+	+	na	+	+
51	IR93283	+	+	-	+	+	na	+	+	+	na	+	+
52	IR93217	+	+	-	+	+	+	+	+	+	+	+	+
53	IR88789	+	+	-	+	+	+	+	+	+	+	+	+
54	IR100923	+	+	-	+	+	+	+	+	+	na	+	+
55	IR86549	+	+	-	+	+	+	+	+	+	na	+	+
56	IR105735	+	+	-	+	+	na	+	+	+	+	+	+
57	IR80600	+	+	-	+	+	na	+	+	+	na	+	+
58	CR10018A	na	+	-	+	+	+	+	+	+	+	+	+
59	IR82979	+	+	-	+	+	na	+	+	+	+	+	+
60	IR106761	+	+	-	na	+	+	+	+	+	+	+	+

S. No.	Accession	*Pup*K-04	***Pup* K-05a	*Pup*K -05b	*Pup*K -20	*Pup*K -29	K-41	K-42	K-43	K-46	K-48	K-52	K-59
61	IR106423	+	+	-	+	+	+	+	+	+	+	+	+
62	IR93285	+	+	-	+	+	+	+	+	+	+	+	+
63	IR93215	+	+	-	+	+	+	+	+	+	+	+	+
64	IR105883	+	+	-	+	+	+	+	+	+	+	+	+
65	IR81989	na	+	-	+	+	+	+	+	+	+	+	+
66	IR86555	+	+	-	+	+	+	+	+	+	+	+	+
67	IR106326	+	+	-	+	+	+	+	+	+	+	+	+
68	IR80600	+	+	-	+	+	+	+	+	+	+	+	+
69	CR10018A	+	+	-	+	+	+	+	+	+	+	+	+
70	IR82989	+	+	-	+	+	+	na	+	+	+	+	+
71	IR106169	+	+	-	na	+	+	+	+	+	+	+	+
72	IR106424	+	+	-	na	+	+	na	+	+	+	+	+
73	IR93202	+	+	-	+	+	+	+	+	+	+	+	+
74	IR93216	+	na	-	+	+	+	+	+	+	na	+	+
75	IR105884	+	na	-	na	+	na	+	+	+	+	+	+
76	IR83810	+	+	-	na	+	na	+	+	na	+	+	+
77	IR86556	+	+	-	na	+	+	+	+	+	+	+	+
78	IR106336	+	+	-	+	+	+	na	+	+	+	+	+
79	IR80610	+	+	-	+	+	+	na	+	+	+	+	+
80	CR100018	+	+	-	+	+	na	na	+	+	+	+	+
81	IR 106290	+	+	-	+	+	+	na	+	+	+	+	+
82	IR106407	+	+	-	+	+	+	+	+	na	+	+	+
83	IR106425	na	+	-	+	+	+	+	+	+	+	+	+
84	IR93203	+	+	-	na	+	na	na	+	na	+	+	+
85	IR105696	+	+	-	na	+	+	+	+	na	+	+	+
86	IR105887	+	+	-	na	+	+	+	+	na	na	+	+
87	IR83811	+	+	-	na	+	na	+	+	+	na	+	+
88	IR86659	+	+	-	na	+	na	na	+	na	na	+	+
89	IR110406	+	+	-	+	+	+	+	+	na	+	+	+
90	IR80660	na	+	-	+	+	+	+	+	na	+	+	+
91	CR100029	+	+	-	+	+	+	+	+	+	+	+	+

S. No.	Accession	*Pup*K-04	***Pup*K-05a	*Pup*K-05b	*Pup*K-20	*Pup*K-29	K-41	K-42	K-43	K-46	K-48	K-52	K-59
92	IR106290	+	+	-	+	+	+	na	na	+	+	+	+
93	IR106427	+	+	-	+	+	+	+	+	+	+	+	+
94	IR80762A	na	+	-	+	+	+	na	na	na	na	+	+
95	IR88818	na	+	-	+	+	+	+	+	+	+	+	+
96	IR104404	na	+	-	+	+	+	+	+	+	na	+	+
97	IR81969	na	+	-	+	+	+	+	+	+	+	+	+
98	IR104459	na	+	-	+	+	+	+	+	+	+	+	+
99	IR89012	+	+	-	+	+	+	+	+	+	+	+	+
100	IR93014	+	+	-	+	+	+	+	+	+	+	+	+
101	CR100006	+	+	-	+	+	+	+	+	+	+	+	+
102	CR100015	+	+	-	+	+	+	+	+	+	+	+	+
103	CR100401	+	+	-	+	+	+	na	na	+	na	+	+
104	IR106503	+	+	-	+	+	+	+	+	+	+	+	+
105	IR113651	+	+	-	+	+	+	+	+	+	+	+	+
106	IR80762B	+	+	-	+	+	+	+	+	+	+	+	+
107	IR99549	+	+	-	+	+	+	+	+	+	+	+	+
108	IR104404A	na	+	-	+	+	+	+	na	na	na	+	+
109	IR101941	na	+	-	+	+	+	+	+	+	na	+	+
110	IR104495	na	+	-	+	+	+	+	+	+	+	+	+
111	IR89020	na	+	-	+	+	+	+	+	+	+	+	+
112	IR93043	+	+	-	+	+	+	+	+	+	na	+	+
113	CR100007	na	+	-	+	+	+	na	na	+	+	+	+
114	CR100035A	+	+	+	+	+	+	+	+	+	+	+	+
115	CR100402	+	+	-	+	+	+	+	+	+	+	+	+
116	IR106504	+	+	-	+	+	+	+	+	na	+	+	+
117	IR99551	+	+	-	+	+	+	+	+	+	+	+	+
118	IR83813	+	+	-	+	+	+	+	+	na	+	+	+
119	IR106149	+	+	-	+	+	+	+	+	+	+	+	+
120	IR104404B	+	+	-	+	+	+	+	+	+	na	+	+
121	IR103850	+	+	-	+	+	+	+	+	na	+	+	+
122	IR104639	+	+	-	+	+	+	+	+	+	+	+	+

**S. No.**	**Accession**	***Pup*K-04**	*****Pup* K-05a**	***Pup*K -05b**	***Pup*K -20**	***Pup*K -29**	**K-41**	**K-42**	**K-43**	**K-46**	**K-48**	**K-52**	**K-59**
123	IR89046	+	+	-	+	+	+	+	+	+	+	+	+
124	IR93048	+	+	+	+	+	+	+	+	+	+	+	+
125	CR100004	+	+	-	+	+	+	+	+	na	+	+	+
126	CR100055	+	+	-	+	+	+	+	+	na	+	+	+
127	IR93137	+	+	-	+	+	+	+	+	+	+	+	+
128	IR106506	+	+	-	+	+	+	+	+	+	+	+	+
129	IR106423	+	+	+	+	+	+	+	+	+	+	+	+
130	IR83814	+	+	+	+	+	+	+	+	na	+	+	+
131	IR106150	+	+	-	+	+	+	+	+	+	+	+	+
132	IR104404C	+	+	-	+	+	+	na	na	+	+	+	+
133	IR104395	+	+	+	+	+	+	+	+	+	+	+	+
134	IR104641	+	+	-	+	+	+	na	na	+	+	+	+
135	IR89223	+	+	-	+	+	+	na	na	+	+	+	+
136	IR93058	+	+	-	+	+	+	+	+	+	+	+	+
137	CR100005	+	+	-	+	+	+	+	+	+	+	+	+
138	CR100375	+	+	-	+	+	+	+	+	+	+	+	+
139	IR100588	+	+	-	+	+	+	+	+	+	na	+	+
140	IR93217	+	+	-	+	+	+	+	+	+	na	+	+
141	IR106349	+	+	-	+	+	+	+	+	+	+	+	+
142	IR106152	+	+	-	+	+	+	+	+	+	na	+	+
143	IR104404D	+	+	-	+	+	+	+	+	na	+	+	+
144	IR104397	+	+	-	+	+	+	+	+	+	na	+	+
145	IR104661	+	+	-	+	+	+	+	+	+	na	+	+
146	IR89224	+	+	-	+	+	+	na	na	na	na	+	+
147	IR93059	+	+	-	+	+	+	+	+	+	+	+	+
148	CR100005B	+	+	-	+	+	+	+	+	na	+	+	+
149	IR80433	+	+	-	+	+	+	+	na	+	+	+	+
150	IR103308	+	+	-	+	+	+	+	+	+	+	+	+
151	IR93219	+	+	-	+	+	na	+	+	na	na	+	+
152	IR105494	+	+	-	+	+	+	+	+	na	na	+	+
153	IR106156	+	+	-	+	+	+	+	+	+	+	+	+

S. No.	Accession	*Pup*K-04	***Pup* K-05a	*Pup*K -05b	*Pup*K -20	*Pup*K -29	K-41	K-42	K-43	K-46	K-48	K-52	K-59
154	IR104404E	+	+	-	+	+	+	+	+	+	+	+	+
155	IR104423	+	+	-	+	+	+	+	+	+	+	+	+
156	IR104712	+	+	-	+	+	+	+	+	+	+	+	+
157	IR89230	+	+	-	+	+	+	+	+	+	+	+	+
158	IR93060	+	+	-	+	+	+	+	+	+	+	+	+
159	CR100006A	+	+	-	+	+	+	+	+	+	+	+	+
160	CR100379A	+	+	-	+	+	na	+	+	na	+	+	+
161	IR100657	+	+	-	+	+	na	na	na	+	+	+	+
162	IR93221	+	+	-	+	+	na	+	+	na	+	+	+
163	IR106343	+	+	-	+	+	+	na	na	+	+	+	+
164	IR106162	+	+	-	+	+	+	+	+	na	+	+	+
165	IR104433	+	+	-	+	+	+	+	+	+	+	+	+
166	IR104424	+	+	-	+	+	+	+	+	+	+	+	+
167	IR104714	+	+	-	+	+	+	+	+	na	+	+	+
168	IR93034	+	+	-	+	+	+	+	na	+	+	+	+
169	IR93062	+	+	-	+	+	+	+	na	+	+	+	+
170	CR100013	+	+	-	+	+	+	+	na	+	+	+	+
171	CR100381	+	+	-	+	+	+	+	na	+	+	+	+
172	IR100678	+	+	+	+	+	+	+	+	+	+	+	+
173	IR93222	+	+	-	+	+	+	+	+	+	+	+	+
174	IR80762	+	+	-	+	+	+	+	+	+	+	+	+
175	IR106161	+	+	-	+	+	+	+	+	+	+	+	+
176	IR104852	+	+	-	+	+	+	+	na	na	+	+	+
177	IR104425	+	+	-	+	+	+	+	+	+	+	+	+
178	IR104716	+	+	-	+	+	+	+	na	na	+	+	+
179	IR89013	+	+	-	+	+	+	+	+	+	+	+	+
180	IR93063	+	+	-	+	+	+	+	+	+	+	+	+
181	CR100013A	+	+	-	+	+	+	+	na	na	+	+	+
182	CR100383	+	+	-	+	+	+	+	+	+	+	+	+
183	Cultivar PR114	+	+	-	+	+	+	+	+	-	+	+	+

S. No.	Accession	***Pup*K-04**	*******Pup*K-05a**	***Pup*K-05b**	***Pup*K-20**	***Pup*K-29**	**K-41**	**K-42**	**K-43**	**K-46**	**K-48**	**K-52**	**K-59**
184	Cultivar PR121	+	+	-	+	+	+	+	+	-	+	+	+
185	Cultivar PR122	+	+	-	+	+	+	+	+	-	+	+	+
186	Cultivar PB3	+	+	-	+	+	+	+	+	+	+	+	+
187	Cultivar VANDANA	+	+	-	+	+	+	+	+	+	+	+	+

***(**+) shows presence of Vandana allele, (-) shows absence of Vandana allele and “na” stands for no amplification. ** PupK-05a: allele 1, PupK-05b: allele 2
