# Supplementary material for: Novel Alleles of Phosphorus-Starvation Tolerance 1 Gene (PSTOL1) from Oryza rufipogon Confers High Phosphorus Uptake Efficiency
Source: Front Plant Sci. 2017 Apr 11;8:509. doi: 10.3389/fpls.2017.00509 (PMC5387083; doi:10.3389/fpls.2017.00509)
Supplement: Supplementary Table S4 — The Prosite analysis of PSTOL1 protein model of Kasalath and Oryza rufipogon accession IRGC 106336. [file Table4.PDF]

Supplementary Table 4: The prosite analysis of *PSTOL1* protein model of Kasalath and *Oryza rufipogon* accession IRGC 106336.

Predicted Features	Kasalath			*Oryza rufipogon* accession IRGC106336		
	Start	Stop	Feature	Start	Stop	Feature
DOMAIN	39	319	Protein kinase	39	136	Tyrosine kinase
NP_BIND	45	53	ATP	45	53	ATP
BINDING	67		ATP	67		ATP
ACT_SITE	166		Proton acceptor	-		**Proton acceptor missing**
